# Serial Transverse Duodenal Enteroplasty in Adults With Ultra-Short Bowel Syndrome: A Case Series

**DOI:** 10.7759/cureus.83145

**Published:** 2025-04-28

**Authors:** Jaime A Ramírez-Arbeláez, Robinson Orjuela, Ana Ramirez, Henry Giraldo, Carlos M Ardila

**Affiliations:** 1 Transplant, Hospital San Vicente Fundación, Rionegro, COL; 2 Surgery, Intestinal Rehabilitation Center, Hospital San Vicente Fundación, Medellin, COL; 3 Surgery, Escuela de Ingeniería de Antioquia, Medellin, COL; 4 Surgery, Universidad del Valle, Cali, COL; 5 Basic Sciences, Facultad de Odontología, Universidad de Antioquia, Medellin, COL

**Keywords:** autologous intestinal reconstruction, duodenal lengthening, intestinal autonomy, intestinal failure, intestinal rehabilitation, parenteral nutrition, serial transverse enteroplasty

## Abstract

Intestinal failure is defined as a reduction in function below the minimum required for adequate absorption of water, electrolytes, and nutrients, necessitating intravenous support. One of the most common etiologies is short bowel syndrome (SBS). For many years, intestinal transplantation was the only surgical option to achieve reduced dependence on parenteral nutrition (PN). However, intestinal lengthening surgery, such as serial transverse enteroplasty (STEP), has proven to be a safe and effective option for select patients with SBS, including those with ultra-short bowel syndrome (USBS) or PN-dependent SBS. Nevertheless, reported cases of STEP in adults are isolated due to the high complexity of the procedure.

We present a case series of three patients with USBS and type III intestinal failure, in whom STEP was performed as part of intestinal rehabilitation. At the time of writing, one patient achieved enteral autonomy, while the other two have significantly reduced their PN requirements (by 33% and 20%, respectively) and improved oral tolerance. Autologous gastrointestinal reconstruction plays a key role in the treatment of intestinal failure secondary to SBS. Among the surgical options, STEP has been successfully used in children, though adult reports remain scarce, particularly for duodenal lengthening (with ≤ two cases reported globally). Postoperative outcomes are favorable, with low morbidity and mortality, offering the possibility of reducing PN dependence and, in select cases, avoiding intestinal transplantation. STEP is a complex but safe and effective procedure for adult patients with SBS, including USBS. It may enable reduced PN dependence, though further studies are needed to define its role in avoiding transplantation.

## Introduction

Intestinal failure is defined as a reduction in function below the minimum necessary for the adequate absorption of water, electrolytes, and nutrients, requiring intravenous support. It has transitioned from being a little-known condition to an active field of work and research. Short bowel syndrome (SBS), defined as having less than 200 cm of small intestine, is the most common etiology [1]. Nowadays, treatment includes a broader therapeutic arsenal beyond parenteral nutrition (PN) and intestinal transplantation [2].

Currently, surgical intervention with autologous gastrointestinal reconstruction is a fundamental pillar in the treatment of intestinal rehabilitation programs [3]. Among these types of surgeries, intestinal lengthening procedures are prominent, including Bianchi’s surgery, with the most commonly used being the serial transverse enteroplasty (STEP), excluding the duodenum [2,3]. The objective of this study is to present three cases of intestinal failure due to ultra-short bowel syndrome (USBS) in adult patients who underwent duodenal STEP as part of their treatment. One patient achieved complete intestinal autonomy, while the other two have improved tolerance to enteral nutrition and are undergoing progressive weaning from PN.

## Case presentation

Case 1

A 27-year-old male patient with no significant medical history presented with massive mesenteric ischemia, requiring extensive intestinal resection, leaving him with the entire duodenum (starting from the first portion) and the descending colon. Nutritional therapy and physical rehabilitation were initiated, and the patient remained dependent on PN with minimal oral intake. He underwent a STEP procedure starting from the first portion of the duodenum, with an anastomosis to the descending colon, resulting in a total of 43 cm of small intestine (including the duodenum). PN and GLP-2 therapy were continued, and 12 months post procedure, the patient has experienced weight gain, improved physical condition, and exercise tolerance. Progressive weaning from PN was initiated, starting at 33 kcal/kg, and currently at 20 kcal/kg, with only a 1 kg weight loss, resulting in a BMI of 19 kg/m². Hematology studies did not lead to a definitive diagnosis, and the patient is on permanent anticoagulation.

Case 2

A 42-year-old female patient with hypothyroidism and hypertension experienced an intestinal volvulus with massive necrosis during pregnancy, requiring resection from the fourth portion of the duodenum to the transverse colon. Nutritional therapy and physical rehabilitation were initiated, and the patient remained dependent on PN with minimal oral intake. She underwent a STEP duodenal procedure starting from the first portion of the duodenum, which required sphincteroplasty of the ampulla of Vater, resulting in a total duodenal length of 21 cm, which was anastomosed to the transverse colon. After the intestinal rehabilitation process, home PN support, and GLP-2 therapy, 10 months post procedure, the patient has achieved weight gain and improved physical condition, and is being weaned off PN, reducing from 33 kcal/kg to 22 kcal/kg without any impact on her weight. Her most recent BMI is 20 kg/m².

Case 3

A 52-year-old male patient with no significant medical history experienced complications after laparoscopic cholecystectomy, resulting in duodenal ulceration and perforation, which required intestinal resections and a Roux-en-Y reconstruction. He developed multiple entero-atmospheric fistulae and intestinal failure, and underwent autologous intestinal reconstruction, leaving him with 35 cm of small intestine (measured from the angle of Treitz), including approximately 10 cm of ileum, an intact ileocecal valve, and a complete colon. Subsequently, the patient developed small bowel necrosis, requiring re-intervention and resolution of complications, leaving him with a duodenum, 20 cm of small intestine, and a complete colon. After physical and nutritional rehabilitation, a duodeno-jejunal STEP lengthening procedure was performed, resulting in a total of 60 cm of small intestine, an intact ileocecal valve, and a complete colon. The patient continues rehabilitation, with weight gain and nutritional improvement through PN and GLP-2 therapy. 14 months post procedure, the patient achieved intestinal autonomy and was successfully weaned off PN.

Description of the procedure

Surgical Procedure (Standard for All Cases)

Preoperative prophylactic IV antibiotics were administered to all patients. Under general anesthesia, an incision was made at the site of the previous scar. After completely separating all the intestinal loops, the length and diameter of the duodenum were measured. An extensive Kocher maneuver was performed to mobilize the posterior wall of the second and third portions of the duodenum and the head of the pancreas, allowing identification of the superior mesenteric vessels. Mobilization of the duodenojejunal flexure was also required to achieve complete exposure of the duodenum. Once the duodenum and the pancreatic head were medially mobilized, sequential firings of the stapler (Endopath Stapler, Ethicon, Endosurgery) were performed on the anterior and posterior anatomical walls of the duodenum perpendicular to the pancreas. The first stapler line was placed anteriorly, starting at the fourth portion of the duodenum. The next stapler firing was performed similarly from the opposite side, creating a channel between the duodenum and the pancreas, repeating as many times as the length of the dilated duodenum allowed (minimum diameter 3 cm). The standard STEP procedure was performed on the dilated jejunum in Case 3. A passage for the mechanical stapler was created between the pancreas and the duodenum by developing holes in the mesentery, as previously detailed [1]. The corners of the staple line were inverted with 4-0 PDS suture. A soft drain was left in place, and all patients began oral intake after seven days following confirmation of the absence of leaks.

Abdominal CT Scan

**Figure 1:**
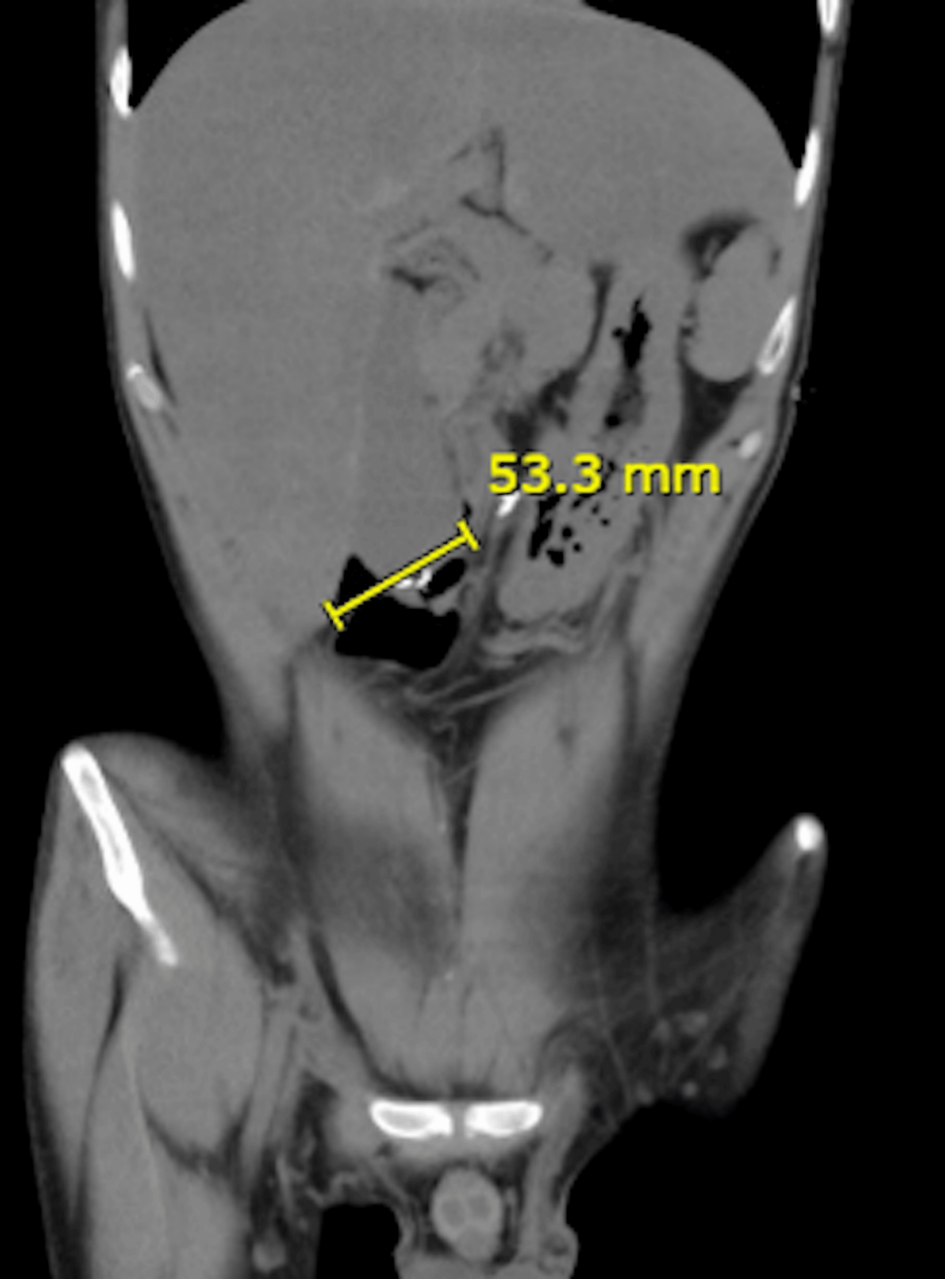
Abdominal CT scan of Case 1 This image shows a coronal view of the abdomen of a 27-year-old male patient with USBS following massive mesenteric ischemia and extensive intestinal resection. The scan reveals significant duodenal dilatation, with a diameter of up to 6 cm, and a complete absence of the small intestine, highlighting the limited absorptive surface necessitating surgical intervention. The yellow line measures the diameter of the significantly dilated duodenum. USBS: Ultra-short bowel syndrome

**Figure 2:**
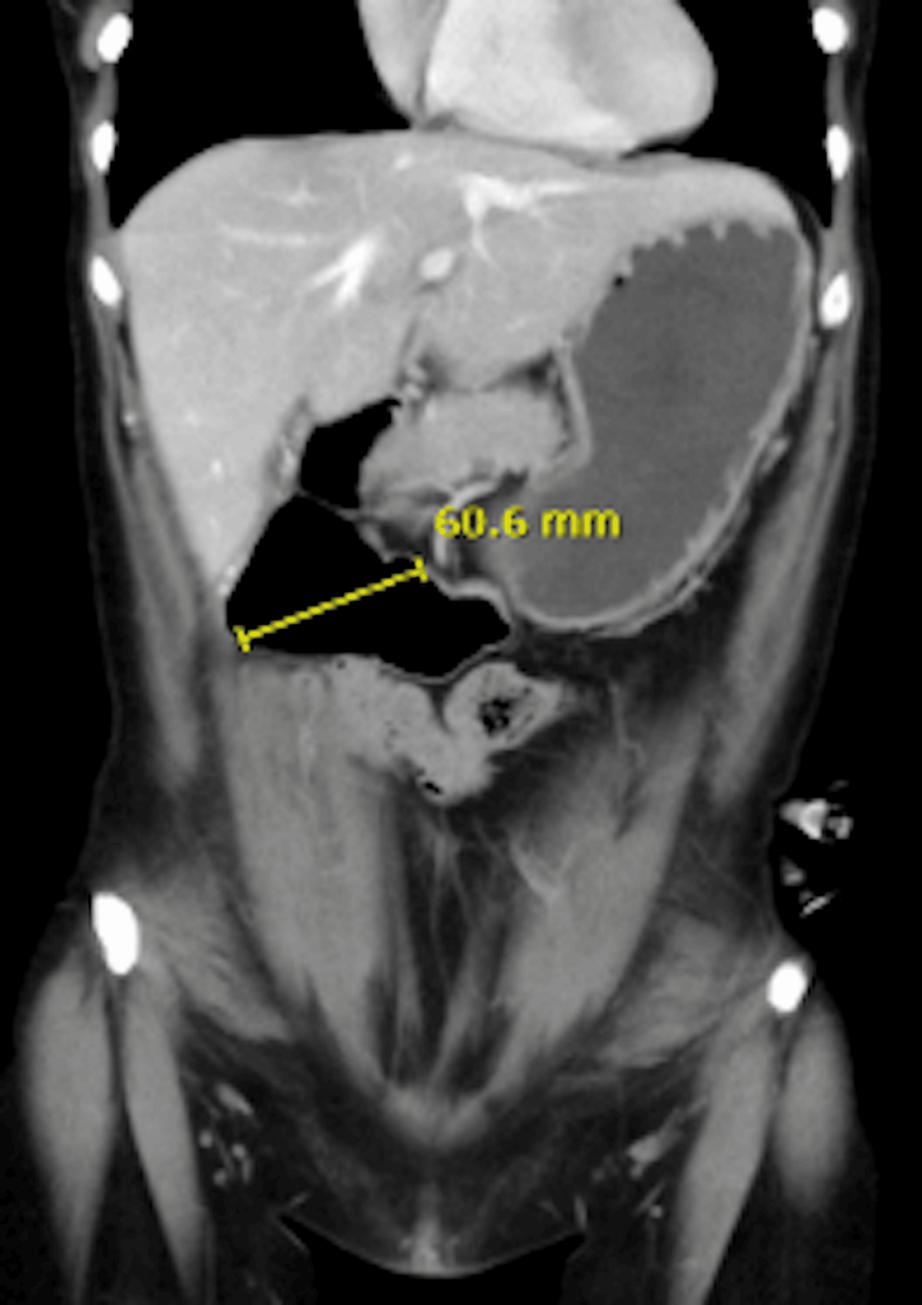
Abdominal CT scan of Case 2 This coronal abdominal CT scan depicts a 42-year-old female patient with USBS after intestinal volvulus and resection during pregnancy. The image demonstrates pronounced duodenal dilatation, measuring up to 6 cm in diameter, with no visible small intestine, underscoring the need for duodenal lengthening to improve nutrient absorption. The yellow line indicates the diameter of the pronounced duodenal dilatation. USBS: Ultra-short bowel syndrome

**Figure 3:**
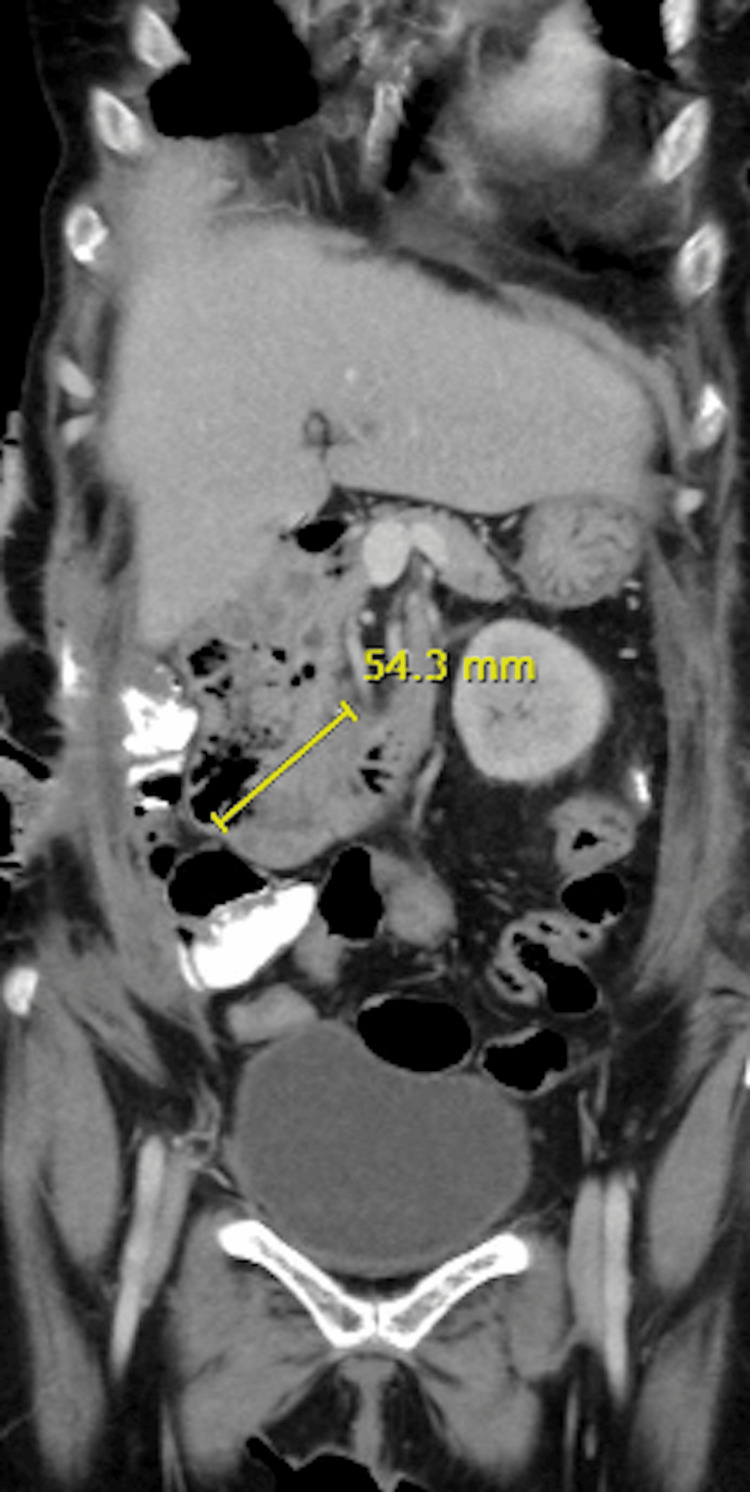
Abdominal CT scan of Case 3 This scan illustrates the abdominal anatomy of a 52-year-old male patient with intestinal failure following complications from laparoscopic cholecystectomy and subsequent resections. It shows a dilated duodenum, up to 6 cm in diameter, and a markedly reduced small intestine, reflecting USBS addressed by the STEP procedure. The yellow line measures the diameter of the dilated duodenum. USBS: Ultra-short bowel syndrome; STEP: Serial transverse enteroplasty

The abdominal CT scan revealed significant duodenal dilatation, measuring up to 6 cm in diameter, and complete absence of the small intestine. The absence of the small intestine suggests that there is little to no absorptive surface available for nutrient uptake, necessitating surgical intervention (Figures 1-3).

Intraoperative Confirmation

**Figure 4:**
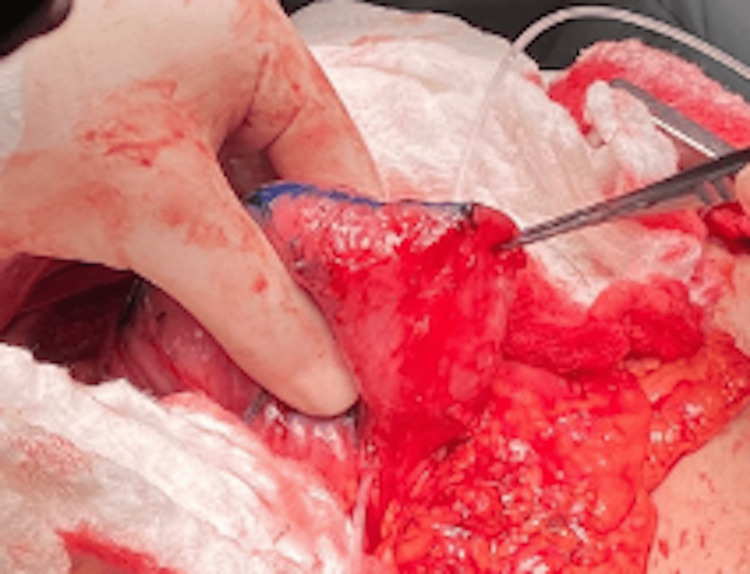
Intraoperative confirmation of duodenal dilatation in Case 2 This intraoperative photograph captures the dilated duodenum of the 42-year-old female patient during the STEP procedure. The image highlights the initial marking of the distal duodenal edge, with the first stapler firing applied from the free surface toward the pancreatic side, initiating the lengthening process. STEP: Serial transverse enteroplasty

Intraoperative findings confirmed the duodenal dilatation. The most distal edge of the free surface of the duodenum was marked, and the first mechanical stapler firing was performed. The stapler was applied from the free surface of the duodenum to the pancreatic side, maintaining a distance of 3 cm from the distal end of the duodenal stump (Figure 4).

Placement of a Nelaton Catheter

**Figure 5:**
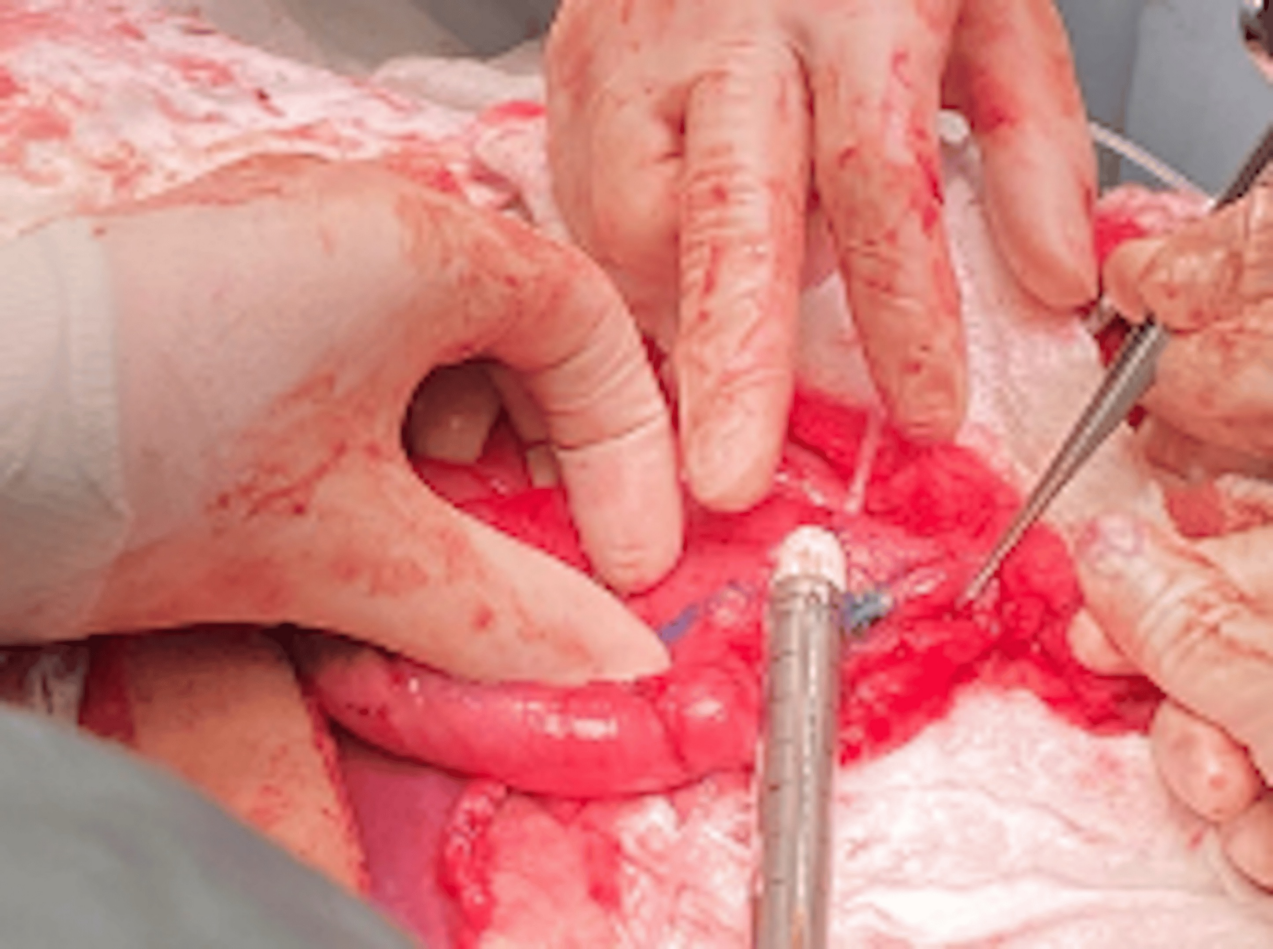
Placement of a Nelaton catheter in Case 2 This intraoperative image shows the insertion of a Nelaton catheter between the duodenum and pancreas in the 42-year-old female patient. The catheter guides the subsequent stapler firing, performed 3 cm from the initial cut in the opposite direction, ensuring precision in creating the zigzag pattern for duodenal lengthening.

**Figure 6:**
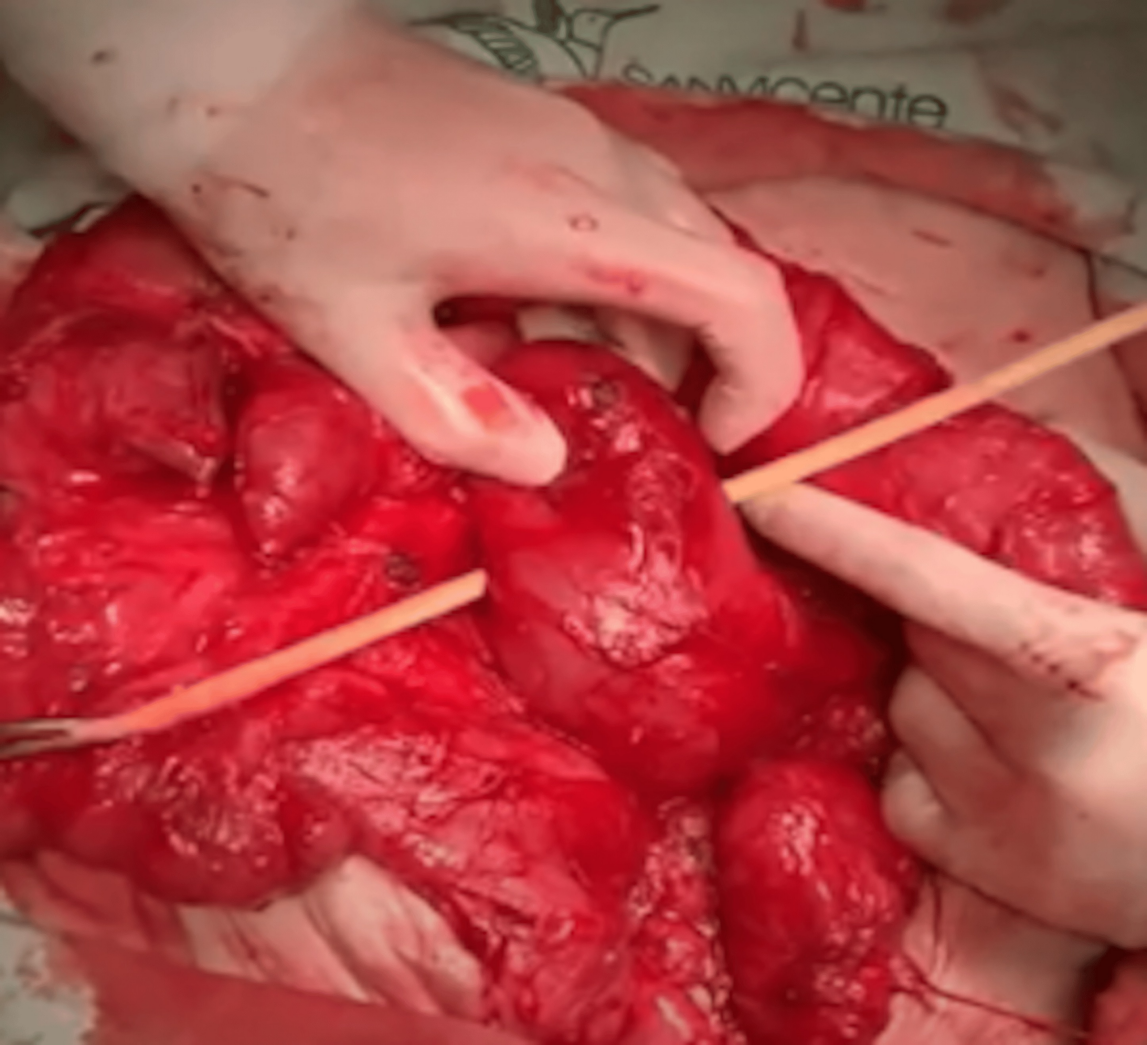
Placement of a Nelaton catheter in Case 3 This photograph depicts the intraoperative placement of a Nelaton catheter in the 52-year-old male patient during the STEP procedure. Positioned between the duodenum and pancreas, the catheter facilitates accurate stapling 3 cm from the prior cut, contributing to the zigzag lengthening of the duodenum. STEP: Serial transverse enteroplasty

A Nelaton catheter was inserted between the duodenum and the pancreas to guide the next cut and intestinal stapling. The stapling was performed 3 cm away and in the opposite direction from the previous stapling. This placement ensured precision in creating the zigzag pattern necessary for adequate intestinal lengthening (Figures 5, 6).

Successive Stapler Firings and Anastomosis

**Figure 7:**
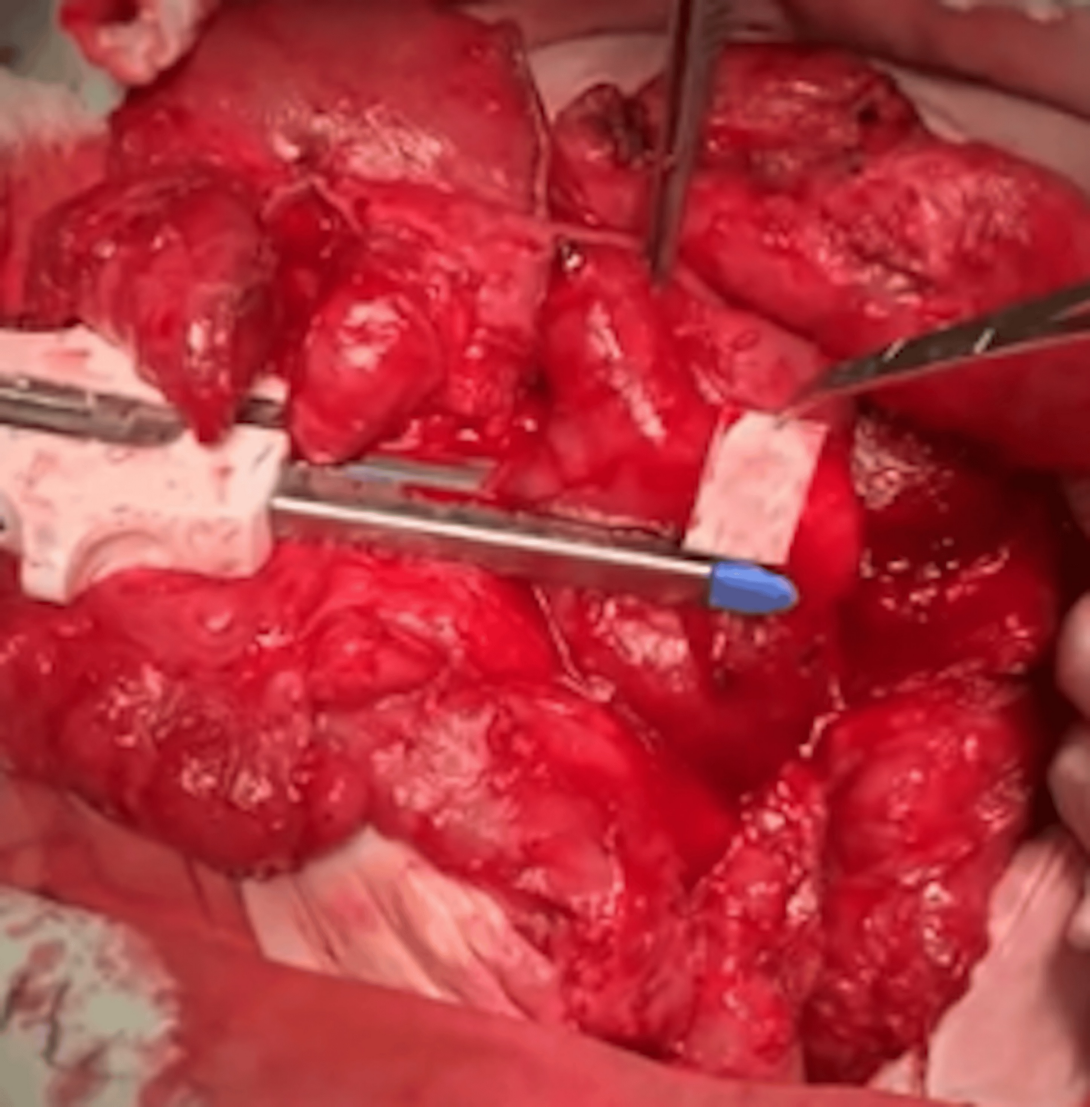
First duodenal cut in Case 3 This intraoperative image illustrates the initial mechanical stapler firing on the dilated duodenum of the 52-year-old male patient. The cut, starting at the fourth portion, is directed perpendicular to the pancreas, marking the beginning of STEP to increase intestinal length. STEP: Serial transverse enteroplasty

**Figure 8:**
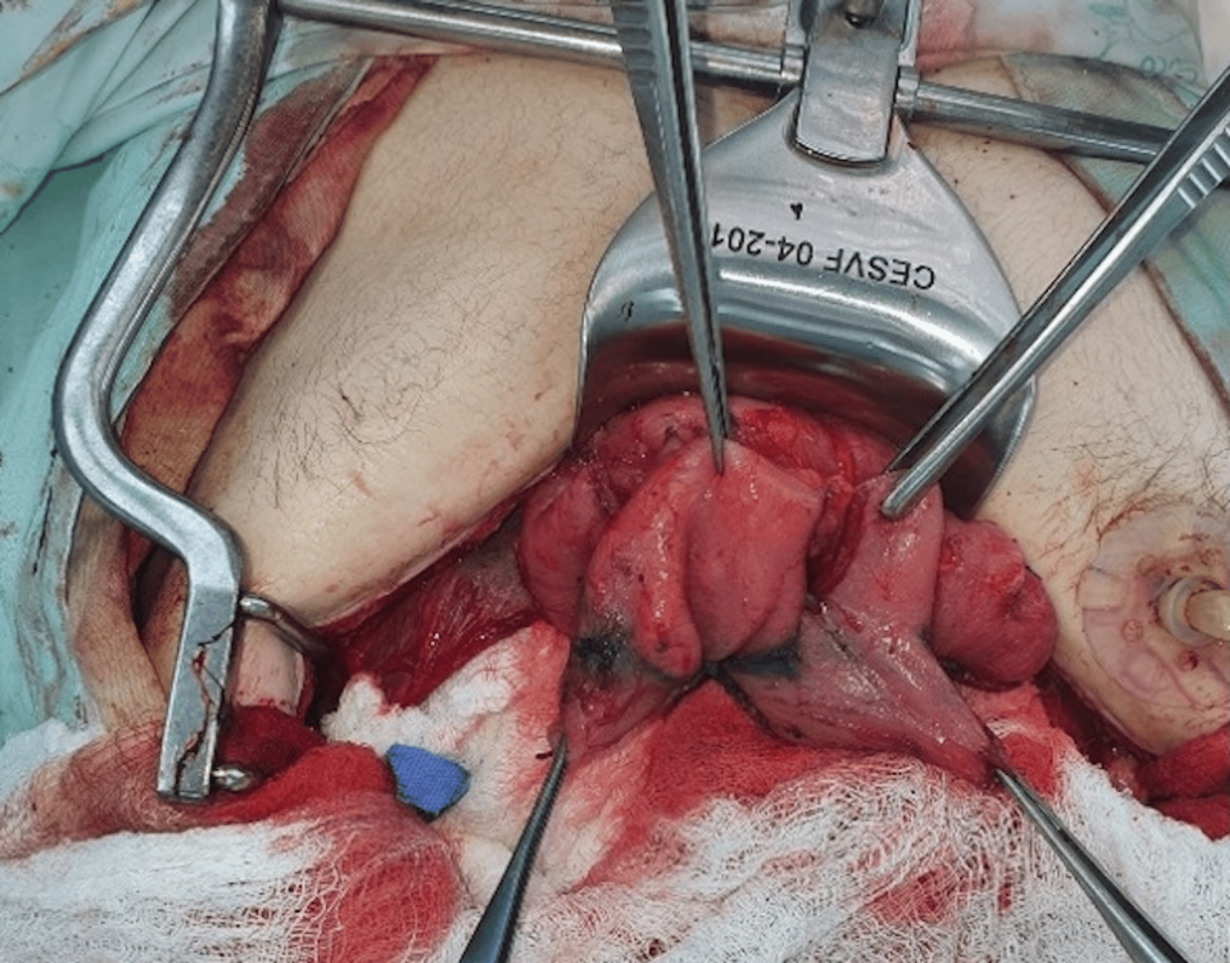
Intraoperative confirmation of duodenal dilatation and initial stapling in Case 1 This photograph shows the dilated duodenum of the 27-year-old male patient during surgery, with the first stapler firing applied anteriorly from the fourth portion. The image captures the early stage of the STEP procedure, aimed at lengthening the 43 cm of remaining small intestine. STEP: Serial transverse enteroplasty

**Figure 9:**
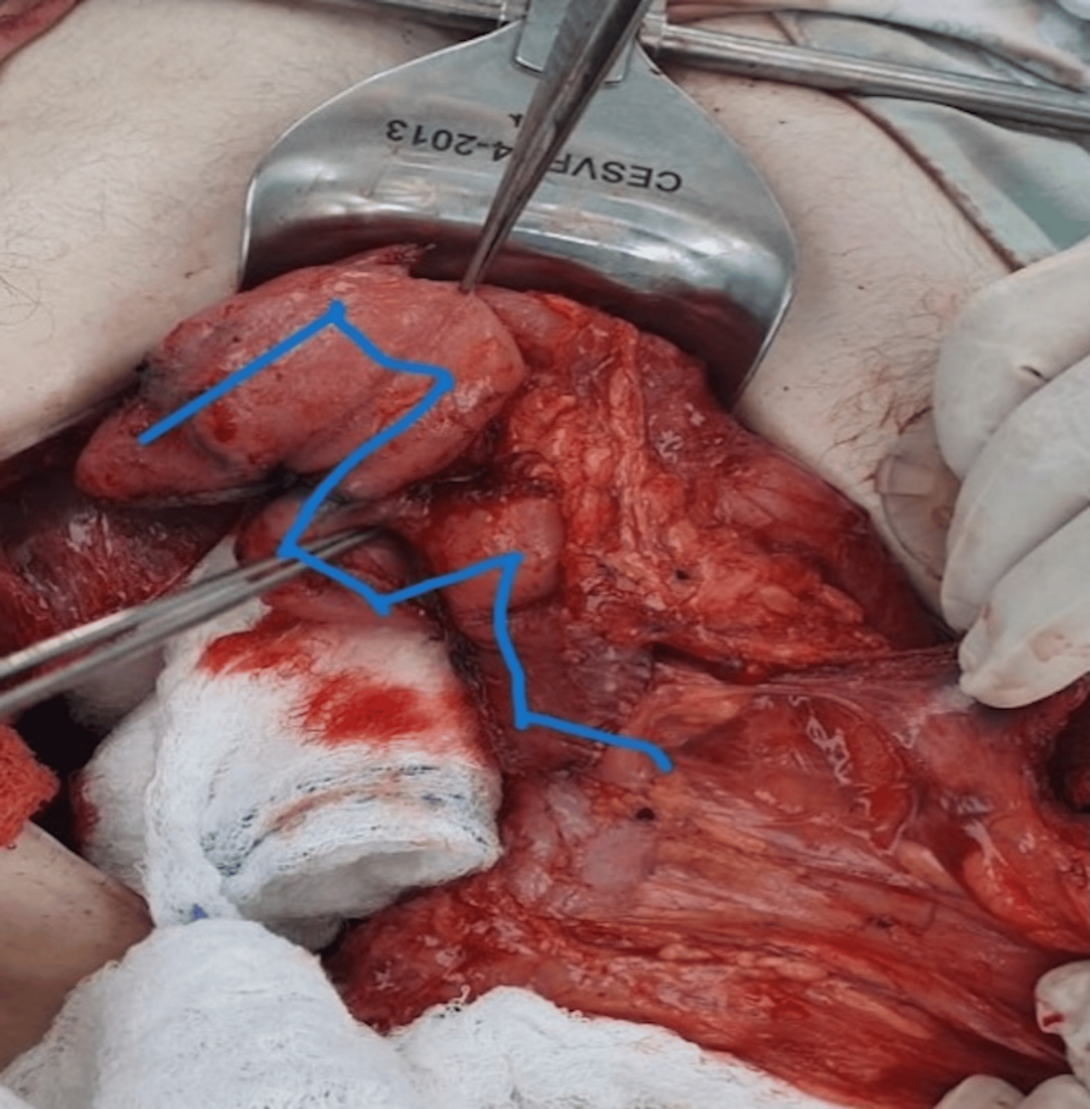
Colon anastomosis in Case 1 This intraoperative image depicts the final step of the STEP procedure in the 27-year-old male patient, showing the anastomosis between the lengthened duodenal stump and the descending colon. This connection restores gastrointestinal continuity, supporting the patient’s progress toward intestinal autonomy. STEP: Serial transverse enteroplasty

**Figure 10:**
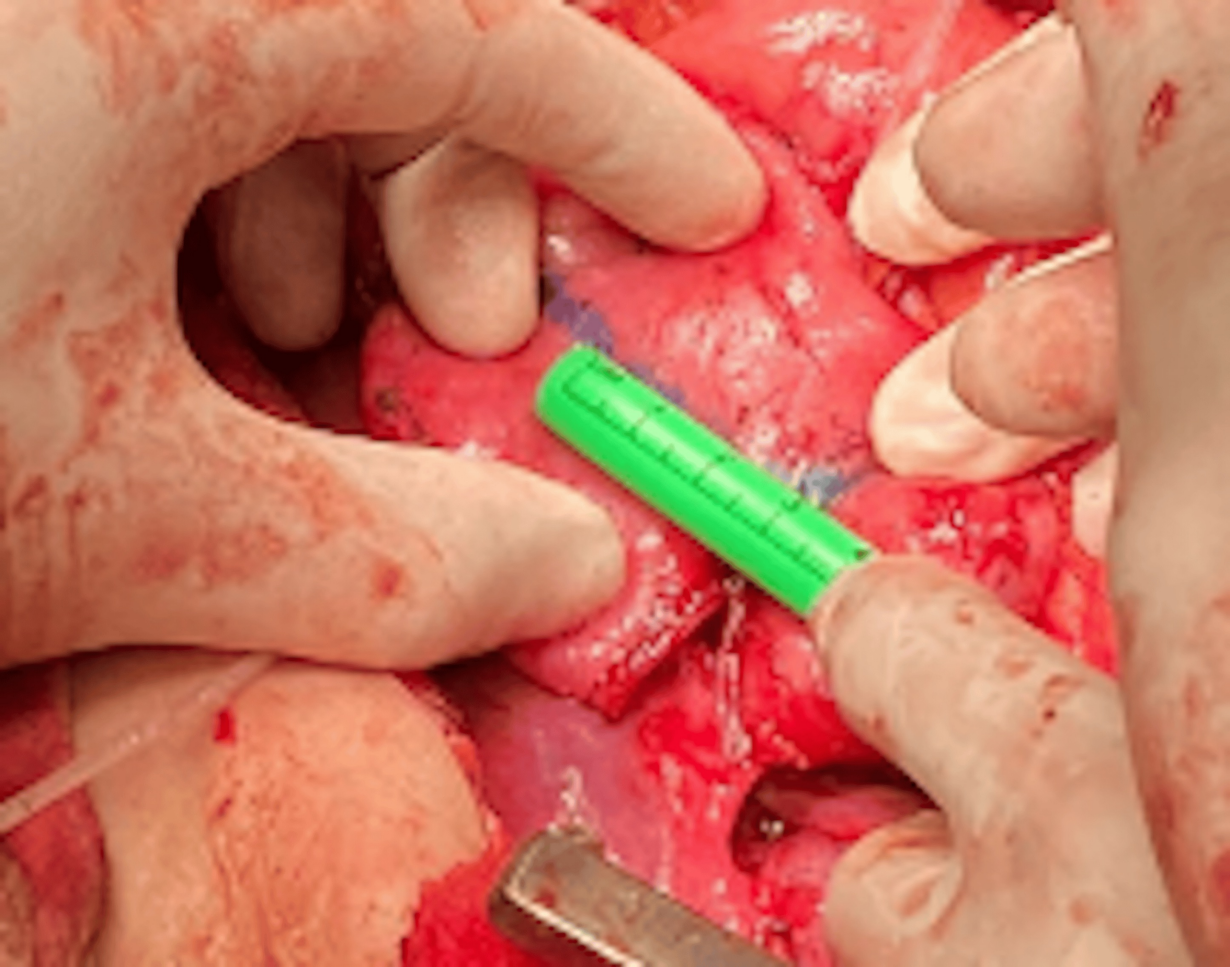
Second cut in Case 2 This photograph illustrates the second stapler firing on the duodenum of the 42-year-old female patient during the STEP procedure. Performed 3 cm from the first cut in the opposite direction, this step continues the zigzag pattern essential for effective duodenal lengthening. STEP: Serial transverse enteroplasty

**Figure 11:**
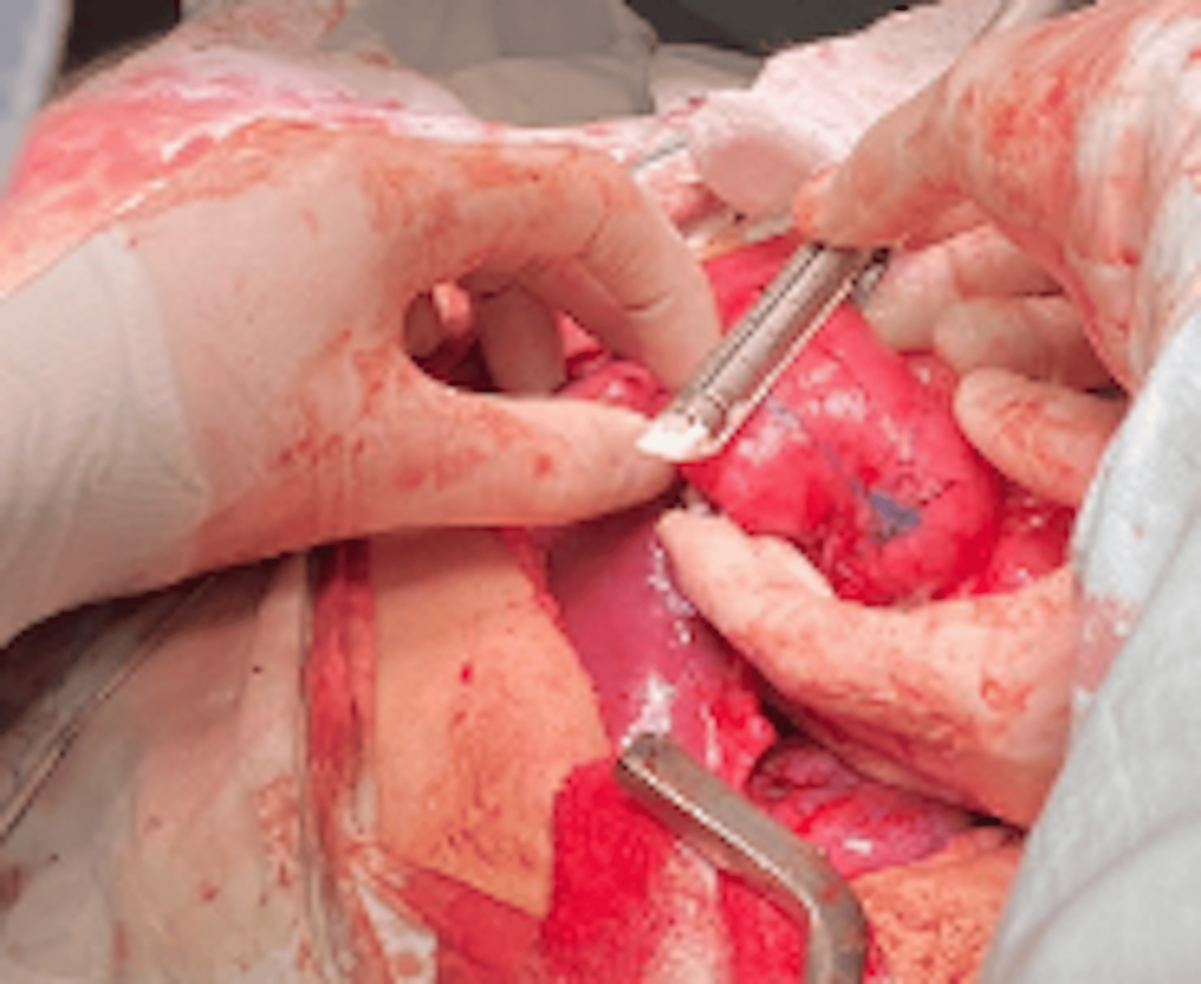
Third cut with reduced duodenal diameter in Case 2 This intraoperative image shows the third stapler firing in the 42-year-old female patient, reducing the duodenal diameter to 2.5 cm. The successive cuts demonstrate the progressive narrowing and lengthening of the duodenum to enhance nutrient absorption.

**Figure 12:**
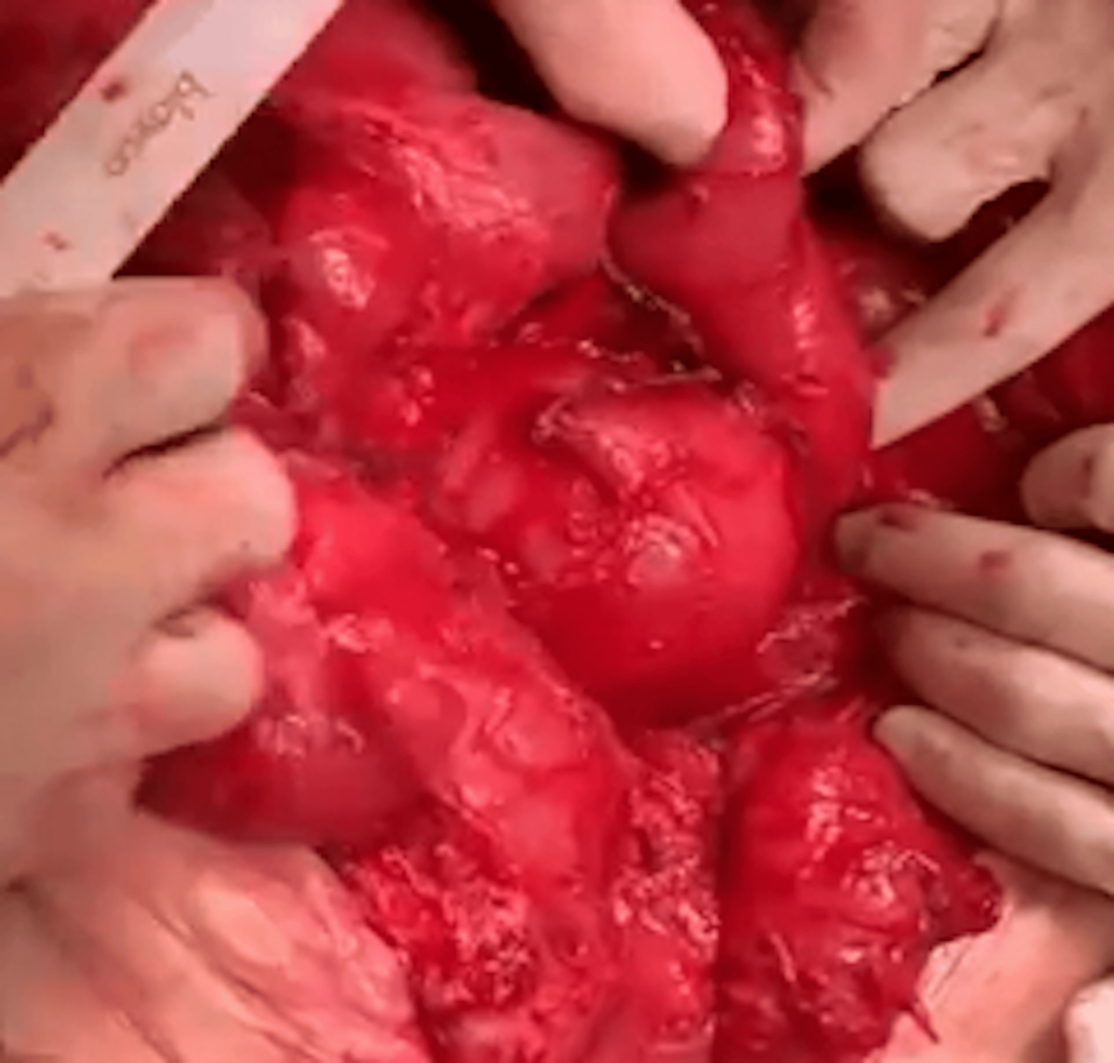
Successive stapler firings at the third duodenal portion in Case 3 This image captures the ongoing STEP procedure in the 52-year-old male patient, showing multiple stapler firings at the third portion of the duodenum. The zigzag pattern is evident, illustrating the technique’s application to increase the intestinal length to 60 cm. STEP: Serial transverse enteroplasty

**Figure 13:**
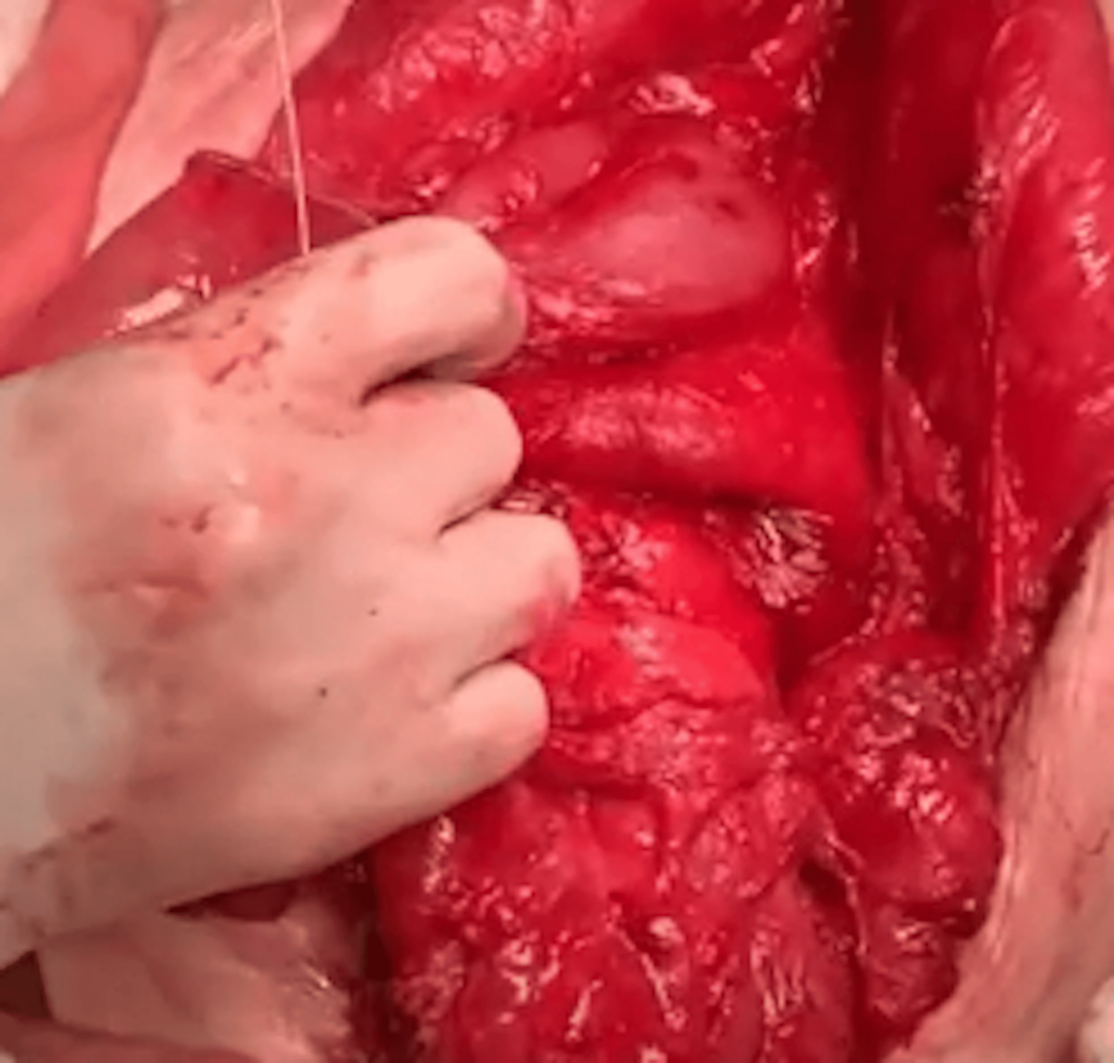
Successive stapler firings at the fourth duodenal portion in Case 3 This photograph depicts the final stages of stapling at the fourth portion of the duodenum in the 52-year-old male patient. The successive firings complete the lengthening process, culminating in an anastomosis with the colon to optimize intestinal function.

The cutting and stapling procedure, measuring 3 cm in length and placed 3 cm from the previous staple line, continued successively in a zigzag pattern until reaching the first portion of the duodenum. Once the lengthening was completed, an intestinal anastomosis was performed between the duodenal stump and the transverse colon, ensuring continuity of the gastrointestinal tract (Figures 7-13).

## Discussion

Intestinal failure can be classified into type I, II, and III based on the duration and functional status of the patient. However, from a pathophysiological perspective, five mechanisms have been identified: extensive intestinal mucosal disease, mechanical intestinal obstruction, intestinal dysmotility, intestinal fistula, SBS (less than 200 cm of small intestine), and USBS (less than 35 cm of small intestine or absence of it) [1,4].

In SBS, health consequences and rehabilitation are directly related to the extent of the available absorptive surface area, in addition to the site and amount of resected intestine. Based on this, three types have been classified: the first includes patients with terminal jejunostomy; the second consists of patients with ileal resection, loss of the ileocecal valve, and partial colon resection where a jejunocolonic anastomosis was achieved; and the third type includes patients with resected and anastomosed loops of small intestine who preserve the ileocecal valve and the entire colon. Each type has different prognoses and responses to treatment, with the last group of patients having a higher likelihood of intestinal rehabilitation [5,6].

Regarding absorptive surface and capacity, half of the colon is equivalent to 50 cm of small intestine, so the presence of this organ in SBS is accepted by multiple authors as a determinant not only for intestinal rehabilitation but also for achieving intestinal autonomy. Buchman et al. indicated that intestinal failure depends on the remnant; those with a poor prognosis include patients presenting with short bowel type 1 and less than 115 cm of small intestine, short bowel type 2 and less than 60 cm of small intestine, or patients with short bowel type 3 and less than 35 cm of small intestine [7].

Until the 1990s, nutritional support (NS) and intestinal transplantation were the only therapeutic options for patients with type 3 intestinal failure [4]. However, the growing enthusiasm of intestinal rehabilitation groups for studying this clinical condition has increased the number of treatment alternatives, such as autologous intestinal reconstruction, intestinal lengthening procedures like STEP, which do not include the duodenum due to its high technical complexity, and hormonal therapies, among others [2,3]. Following the publication of Abu-Elmagd et al.'s study in 2019, surgical management through autologous reconstruction of the gastrointestinal tract has become the fundamental treatment for patients with intestinal failure [3]. This reconstruction aims to optimize the patient's native intestinal tissue and consists of multiple procedures, including fistula closure, restoration of intestinal continuity, management of dilated loops, and intestinal lengthening [8].

Among the techniques for intestinal lengthening, the Bianchi technique, also known as longitudinal intestinal lengthening techniques, has been the most widely used [9]. In 2003, Jones et al. presented STEP in porcine models, achieving an increase in intestinal length of 68% [10]. All animals gained weight, no mortalities were reported, and autopsies showed that the intestine corrected the zigzag pattern resulting from the surgery, returning to a shape similar to its usual form. Subsequent animal studies have demonstrated no alterations in phase III of the migrating motor complex, sustained weight gain, intestinal lengthening, and an increase in jejunal villus height [10].

STEP is considered technically easier to perform, provided that the duodenum is not included, and has the advantages of not communicating the intestinal lumen with the abdominal cavity, thereby reducing the risk of infection and postoperative collections. It allows for the elimination of dysfunctional intestinal dilation while preserving the intestinal mucosa, does not require uniform intestinal dilation, and requires minimal manipulation of the mesentery, which helps preserve the organ's vascularization. It can be performed in the duodenum and does not necessitate a minimum remnant of intestine, although a loop diameter of at least 3.5 to 4 cm is required [9-11]. This technique can be performed in patients with prior STEP or Bianchi procedures, leading to its global adoption and increasing use [12].

In the pediatric population, both lengthening techniques have been performed with satisfactory results, achieving enteral autonomy in up to 40-50% of patients and reducing NS by 25-50% within two years post-procedure. However, as previously mentioned, the Bianchi technique has fallen out of favor due to its complexity and potential complications, with STEP now being performed more frequently [13]. Recent data from the International STEP Data Registry indicate an 11% mortality rate, with none of the patients requiring transplantation, 55% achieving intestinal autonomy, and 14% improving enteral tolerance without reaching autonomy [12].

Despite being a safe technique, there are still few case reports where STEP is performed on the duodenal portion of the intestine due to its technical complexity. It is known that most patients with USBS present with duodenal dilation associated with the dilation of the remnant intestine [14]. This dilation interferes with the adaptation process, facilitating alkaline reflux, dysmotility, stasis, and bacterial overgrowth, leading to mucosal inflammation that impedes digestion and nutrient absorption. To avoid these complications, correcting this dilation is necessary, which is useful not only for increasing intestinal length [14,15] but also for increasing luminal transit time and improving nutrient absorption [15]. However, its anatomical relationships complicate its execution due to proximity to the pancreas, common vascular supply, and absence of mesentery, along with the close relationship with the bile and pancreatic ducts [14].

Regarding duodenal lengthening by STEP, Bueno et al. reported three cases in the pediatric population where this procedure was performed; two of the three patients achieved intestinal autonomy [15]. Although this is a limited number of patients, the results of this procedure have been effective in managing USBS with duodenal dilation [15]. For adult duodenal lengthening, global literature is scarce, with only two reported cases of duodenal STEP. One was of an 18-year-old patient with 32 cm of residual intestine from the pylorus to the entero-colic anastomosis, performed due to recurrent episodes of D-lactic acidosis [14]. The other case involved a 25-year-old patient with a history of midgut volvulus, from whom a quarter of the duodenum, all of the jejunum, ileum, and ascending colon were resected [16]. Duodenal lengthening was performed with seven sequential transverse applications, resulting in a lengthening from 30 cm to 83 cm. Follow-up continued for 24 months, during which the patient achieved mixed nutrition [16].

This is the first successful report of surgical management with duodenal lengthening in adult patients with USBS, achieving enteral autonomy and reducing the need for PN and subsequent intestinal transplantation in the short term. In the other two patients, a reduction of up to 33% in their parenteral intake was achieved in just one year, without affecting their weight or hydroelectrolytic status. Regarding the surgical technique, no changes were made to the standard procedure, creating small windows between the duodenum and the pancreas as originally done in the mesentery. Although one case presented injury to the ampulla of Vater, sphincteroplasty was performed, and the patient remains free of biliary complications.

## Conclusions

Serial transverse duodenal enteroplasty is a complex but safe procedure for adult patients with USBS. In this short-term follow-up (up to 24 months), our case series demonstrates that duodenal STEP reduces dependence on PN (33% reduction in two patients) and may mitigate the need for eventual intestinal transplantation by promoting intestinal adaptation; achieved enteral autonomy in one patient, highlighting its potential as a bridge therapy in intestinal rehabilitation; and requires careful technical execution due to anatomical constraints but has acceptable complication rates. While these results are promising, longer-term studies are needed to evaluate durability and transplantation-free survival.
